# Beyond bacteremia: clinical phenotypes and determinants of mortality in hospitalized adults with community-acquired urinary tract infection

**DOI:** 10.3389/fmed.2026.1754663

**Published:** 2026-02-11

**Authors:** Cihan Semet, Yusuf Görgülü

**Affiliations:** 1 Department of Infectious Diseases and Clinical Microbiology, Faculty of Medicine, Bandirma Onyedi Eylül University, Bandirma, Türkiye; 2 Department of Microbiology, Faculty of Medicine, Bandirma Onyedi Eylül University, Bandirma, Türkiye

**Keywords:** antimicrobial resistance, bacteremia, community-acquired urinary tract infection, C-reactive protein, mortality, procalcitonin, qSOFA, risk factors

## Abstract

**Background:**

Community-acquired urinary tract infection (CA-UTI) is a leading source of bacteremia and sepsis in adults, yet the determinants of bloodstream invasion and short-term mortality remain incompletely defined. In particular, the relative contributions of age, comorbidity, antimicrobial resistance, and acute organ dysfunction are uncertain.

**Methods:**

We conducted a retrospective cohort study of consecutive adults hospitalized with CA-UTI at a tertiary hospital in Türkiye between January 2023 and June 2025. Clinical, laboratory, microbiological, and outcome data were abstracted. Multivariable logistic regression was used to identify predictors of concomitant bacteremia and 30-day all-cause mortality in the overall cohort and in the bacteremic subgroup. An eight-item unweighted risk-factor count score (male sex, short symptom duration, prior extended-spectrum β-lactamase–producing and carbapenem-resistant organisms colonization or infection, Charlson Comorbidity Index (CCI) ≥ 2, diabetes mellitus, qSOFA ≥ 2, C-reactive protein > 100 mg/L, procalcitonin ≥ 0.5 ng/mL) was derived to predict bacteremia, and its discrimination was assessed with ROC analysis.

**Results:**

Among 358 adults with CA-UTI, 117 (32.7%) had concomitant bacteremia. In the multivariable model, independent predictors of bacteremia included male sex, shorter symptom duration, prior extended-spectrum β-lactamase–producing and carbapenem-resistant organisms colonization or infection, CCI ≥ 2, diabetes mellitus, qSOFA ≥ 2, C-reactive protein > 100 mg/L, and procalcitonin ≥ 0.5 ng/mL. The eight-item risk-factor count showed a strong gradient in bacteremia prevalence (3.8% with 0–1 factors, 24.7% with 2–3, and 75.0% with ≥ 4) and good discrimination (AUROC 0.83 for the full model and 0.80 for the simplified score; DeLong p ≈ 0.3). Overall, 30-day mortality was 6.1% (22/358) and higher in bacteremic than non-bacteremic CA-UTI (9.4% vs. 4.6%). In the whole cohort, both bacteremia and qSOFA score ≥ 2 were independent predictors of 30-day mortality. Within the bacteremic subgroup, qSOFA score ≥ 2 was identified as the sole independent predictor.

**Conclusion:**

In adults hospitalized with CA-UTI, bacteremia identifies a high-risk clinical phenotype but is not the sole determinant of prognosis. Bacteremia is a marker of severity, whereas organ dysfunction, as captured by qSOFA, is the main driver of mortality. A simple eight-item bedside score demonstrated promising performance in predicting bacteremia but requires external validation before its routine use in clinical triage.

## Introduction

Urinary tract infections (UTIs) are among the most common community bacterial infections and remain a leading source of bloodstream infections in adults (1, 2). Community-acquired bacteremia of urinary origin contributes substantially to global morbidity and mortality (3, 4). The presence of bacteremia in UTI generally signals more severe disease and is associated with higher risks of sepsis, septic shock, and intensive care requirements (1). Whether bacteremia independently worsens prognosis in UTIs remains a matter of debate. In generally healthy populations, available data suggest that bacteremia does not meaningfully alter clinical outcomes (5). In contrast, studies in adults with complicated UTIs have yielded inconsistent results: some report higher rates of serious complications among bacteremic cases, whereas others find no significant differences in hard outcomes such as mortality (1, 6). This inconsistency highlights the need to clarify the independent effect of bacteremia on disease severity and prognosis across distinct patient subgroups. Identifying risk factors for bacteremia in community-acquired UTIs (CA-UTIs) is therefore critical for early recognition and targeted management of high-risk patients. Prior work highlights several clinical predictors, particularly markers of systemic inflammation and severity, including high fever (≥ 39°C), hemodynamic instability, and elevated infection biomarkers such as procalcitonin, which have each been associated with bloodstream invasion in UTI (7, 8). Advanced age is a consistently identified risk factor for bacteremia, mainly attributable to the higher burden of comorbidities and the increased frequency of atypical clinical presentations in older adults (9). Additionally, male sex, structural urinary tract abnormalities, and recent exposure to healthcare settings may further increase the risk of developing bacteremia (10). From a microbiological perspective, *Escherichia coli*, a Gram-negative bacillus within Enterobacterales, remains the predominant etiologic agent in both uncomplicated and bacteremic urinary tract infections (11). In complicated infections, however, the etiologic spectrum broadens, and other Gram-negative bacilli, Gram-positive organisms, and yeasts are also commonly implicated (12). Given the rising antimicrobial resistance among community-acquired uropathogens and our study’s inclusive approach to all urinary tract infection–causing organisms, we aim to comprehensively assess both host and microbiological determinants.

Although UTIs are highly prevalent, detailed data on community-acquired bacteremic UTIs in adults remain limited. The existing literature has primarily focused on specific subpopulations, leaving broader epidemiological and clinical insights incomplete. There is an ongoing need for community-based studies to identify patients at the highest risk for bacteremia and to clarify its impact on key clinical outcomes, including sepsis severity, intensive care unit (ICU) admission, and mortality. To address this knowledge gap, we conducted a comparative analysis of adult patients treated at our institution with community-acquired bacteremic versus non-bacteremic UTIs. Our primary objective was to delineate the clinical and biological determinants of concomitant bacteremia in adults hospitalized with community-acquired urinary tract infection and to quantify its independent association with short-term clinical outcomes. Secondary objectives were to compare the clinical course of bacteremic versus non-bacteremic CA-UTI and to derive a simple bedside risk score based on admission variables to support early risk stratification at presentation.

Our findings may contribute to a better understanding of bacteremic UTIs in community settings and could help with timely recognition of higher-risk patients and the development of targeted management strategies.

## Materials and methods

### Study design and setting

We conducted a retrospective cohort study at a tertiary university hospital in Türkiye, including consecutive adults admitted with a clinical diagnosis of UTI between 1 January 2023 and 30 June 2025. For each patient, only the first eligible episode within the study period was included.

### Participants and eligibility criteria

Adults (≥ 18 years) admitted with UTI and with a urine culture obtained within 24 h of presentation or admission were screened. We excluded episodes that were hospital-acquired (onset > 48 h after admission), transfers after more than 48 h in another acute-care hospital, cases with an alternative non-urinary source of bacteremia, discordant polymicrobial blood cultures, and episodes with missing key clinical or microbiological data. Blood culture isolates deemed contaminants according to institutional criteria (e.g., typical skin commensals isolated from a single bottle without compatible clinical findings) were excluded from the definition of bacteremia.

### Definitions

Bacteremia was defined as at least one peripheral blood culture positive for a pathogen consistent with a urinary source within 48 h of hospital arrival. CA-UTI was defined as an infection with onset in the community or within 48 h after admission, together with a compatible urinalysis and/or a positive urine culture. Recent healthcare exposure was defined as hospitalization or residence in a long-term care facility within the preceding 90 days. Prior extended-spectrum β-lactamase/carbapenem-resistant organism (ESBL/CRO) status reflected documented colonization or infection within the previous 12 months.

### Severity, comorbidity, and functional status assessment

Severity and functional status were assessed using the Pitt bacteremia score (13), the quick Sequential Organ Failure Assessment (qSOFA; dichotomized as < 2 vs. ≥ 2), sepsis defined as a Sequential Organ Failure Assessment (SOFA) score ≥ 2 (14), the Charlson Comorbidity Index (CCI; categorized as 0, 1, or ≥ 2) (15), and the Barthel Index (BI; < 40, 40–60, or > 60) (16). Dichotomization thresholds for key predictors were selected *a priori* based on established literature and clinical practice: age ≥ 65 years and CCI ≥ 2 were used to capture older adults and patients with a higher comorbidity burden (17, 18), qSOFA ≥ 2 was chosen according to the Sepsis-3 proposal as a marker of high risk for adverse outcomes, and C-reactive protein (CRP) > 100 mg/L and procalcitonin (PCT) ≥ 0.5 ng/mL were selected in line with studies and guideline-driven algorithms associating these thresholds with severe bacterial infection and sepsis rather than localized or low-risk disease (19, 20). Short symptom duration was defined as symptom duration ≤ 3 days before admission (21). This prespecified cutoff was selected to improve clinical interpretability at the bedside and to facilitate the development of a parsimonious risk stratification tool, while acknowledging that dichotomization may attenuate statistical power and limit the assessment of potential dose–response relationships.

### Calculation of qSOFA and SOFA

For all analyses, qSOFA was calculated at the time of hospital presentation using the first set of recorded vital signs (systolic blood pressure, respiratory rate, and Glasgow Coma Scale) in the emergency department or at direct ward admission. SOFA scores were calculated according to the original definitions, using the most abnormal value for each organ system documented during the first 24 h after presentation (emergency department and ward), and are referred to as admission SOFA scores. For patients managed outside the ICU, the same approach was applied using routinely collected laboratory and physiological data; when arterial blood gas measurements were not available, the respiratory component was derived from peripheral oxygen saturation and the use of supplemental oxygen, and in the absence of documented hypoxemia or oxygen therapy the respiratory SOFA score was assumed to be 0.

### Microbiology and antimicrobial susceptibility testing

Blood cultures (when feasible, obtained before the administration of antibiotics) and quantitative urine cultures were processed according to institutional protocols. Organism identification was performed using the VITEK 2 automated system, and antimicrobial susceptibilities were interpreted according to contemporaneous EUCAST breakpoints.

### Definition of appropriate empirical therapy

Appropriate empirical therapy was defined as the administration, within the first 24 h after hospital presentation, of at least one systemic antibiotic with documented *in vitro* activity against the index uropathogen according to EUCAST breakpoints (22, 23).

### Statistical analysis

Statistical analyses were performed using IBM SPSS Statistics, version 29 (IBM Corp., Armonk, NY, United States). Normality of continuous variables was assessed using the Shapiro–Wilk test. Continuous variables are reported as mean (standard deviation, SD) and were compared using Student’s *t*-test or the Mann–Whitney U test, as appropriate; categorical variables are presented as counts and percentages and were compared using the χ^2^ test or Fisher’s exact test. All tests were two-sided, and a *p* < 0.05 was considered statistically significant. Analyses were conducted on complete cases; no imputation of missing data was performed. To identify predictors of concomitant bacteremia, we first performed univariable logistic regression for each candidate variable; those with a *p* < 0.10 in univariable analyses or judged to have strong clinical relevance were then entered into a multivariable logistic regression model with backward likelihood-ratio selection, with age and sex forced to remain in the model. For the 30-day mortality analysis in the overall CA-UTI cohort, we constructed a parsimonious multivariable logistic regression model including bacteremia, age ≥ 65 years, CCI ≥ 2, and qSOFA ≥ 2 as pre-specified predictors based on prior literature and clinical relevance, in order to respect the limited number of deaths and reduce the risk of overfitting. In the bacteremic subgroup, we additionally fitted an exploratory multivariable model including age ≥ 65 years, CCI ≥ 2, and qSOFA ≥ 2 as predictors of 30-day mortality; given the low number of deaths (*n* = 11), this analysis was considered hypothesis-generating and at risk of overfitting. Results of the logistic regression analyses are reported as adjusted odds ratios (aORs) with 95% confidence intervals (CIs).

### Development of the bedside bacteremia risk score and ROC analysis

To derive a simple bedside risk score for predicting bacteremia, we selected eight binary predictors from the final multivariable bacteremia model and clinically relevant variables: male sex, short symptom duration before admission, prior ESBL/CRO colonization or infection within 12 months, CCI ≥ 2, diabetes mellitus, qSOFA ≥ 2, CRP > 100 mg/L, and PCT ≥ 0.5 ng/mL. Each risk factor present contributed one point to an unweighted additive risk-factor count (range 0–8), which was then categorized for descriptive purposes as 0–1, 2–3, or ≥ 4 risk factors. Discriminative performance for bacteremia was evaluated using receiver-operating characteristic (ROC) curve analysis. We generated ROC curves and calculated the area under the ROC curve (AUROC) with 95% CIs for both the full multivariable bacteremia model (using predicted probabilities) and the simplified risk-factor count. AUROCs were compared using the non-parametric DeLong method. Because the score was derived and evaluated within the same cohort and no resampling-based internal validation (e.g., bootstrapping or cross-validation) was performed, the reported AUROC values represent apparent performance and may overestimate accurate discrimination.

### Ethics approval

The study was approved by the Bandırma Onyedi Eylül University Non-Interventional Clinical Research Ethics Committee (Decision No. E-67961857, 07 May 2025). The requirement for informed consent was waived due to the retrospective design and the use of de-identified data.

## Results

### Study population and baseline characteristics

A total of 358 adults hospitalized with CA-UTI were included in the analysis; 117 (32.7%) had concomitant bacteremia and 241 (67.3%) had non-bacteremic CA-UTI (Table 1). The mean age of the cohort was 64.9 ± 16.6 years and did not differ between groups, whereas female sex was less frequent among patients with bacteremia (44.4% vs. 59.8%; p = 0.006). Bacteremic patients presented after a shorter duration of symptoms (3.12 ± 1.60 vs. 4.29 ± 2.09 days; *p* < 0.001) and more often had a history of hospitalization within the previous 90 days (26.5% vs. 17.0%; *p* = 0.036) and prior ESBL/CRO colonization or infection within 12 months (15.4% vs. 4.6%; *p* < 0.001).

**TABLE 1:** Baseline characteristics, comorbidities, clinical presentation, laboratory findings, microbiology, treatment, and outcomes of adults hospitalized with community-acquired urinary tract infection, stratified by bacteremia status.

Variable	All patients (*n* = 358)	Non-bacteremic CA-UTI (*n* = 241)	Bacteremic CA-UTI (*n* = 117)	*p*-value
**Demographics and prior exposures**				
Age, years, mean ± SD	64.88 ± 16.63	64.70 ± 16.21	65.25 ± 17.54	0.805
Female sex, n (%)	196 (54.7)	144 (59.8)	52 (44.4)	0.006
Long-term care facility residence, n (%)	54 (15.1)	34 (14.1)	20 (17.1)	0.459
Symptom duration before admission, days, mean ± SD	3.91 ± 2.02	4.29 ± 2.09	3.12 ± 1.60	**< 0.001**
Previous hospitalization ≤ 90 days, n (%)	72 (20.1)	41 (17.0)	31 (26.5)	**0.036**
Prior ESBL/CRO colonization or infection ≤ 12 months, n (%)	29 (8.1)	11 (4.6)	18 (15.4)	**< 0.001**
**Comorbidities and functional scores**				
CCI, mean ± SD	2.18 ± 1.37	2.10 ± 1.35	2.36 ± 1.40	0.098
Diabetes mellitus, n (%)	91 (25.4)	43 (17.8)	48 (41.0)	**< 0.001**
Hemodialysis, n (%)	8 (2.2)	0 (0.0)	8 (6.8)	**< 0.001**
Dementia, n (%)	21 (5.9)	8 (3.3)	13 (11.1)	**0.003**
Solid organ malignancy, n (%)	13 (3.6)	2 (0.8)	11 (9.4)	**< 0.001**
BI, mean ± SD	72.18 ± 21.33	72.80 ± 21.22	70.90 ± 21.58	0.400
Pitt bacteremia score at presentation, mean ± SD	1.65 ± 1.51	1.22 ± 1.15	2.56 ± 1.74	**< 0.001**
**Clinical findings at admission**				
Fever > 38°C, n (%)	220 (61.5)	137 (56.8)	83 (70.9)	**0.010**
Heart rate > 120 beats per minute, n (%)	63 (17.6)	32 (13.3)	31 (26.5)	**0.002**
Systolic blood pressure < 90 mmHg, n (%)	49 (13.7)	19 (7.9)	30 (25.6)	**< 0.001**
qSOFA ≥ 2, n (%)	26 (7.3)	11 (4.6)	15 (12.8)	**0.005**
Sepsis at admission (SOFA ≥ 2), n (%)	120 (33.5)	61 (25.3)	59 (50.4)	**< 0.001**
**Laboratory findings at admission**				
White blood cells (/mm^3^), mean ± SD	8061.91 ± 2251.81	7547.66 ± 2085.30	9127.16 ± 2218.45	**< 0.001**
Neutrophil-to-lymphocyte ratio, mean ± SD	6.09 ± 4.34	5.45 ± 3.69	7.41 ± 5.23	**< 0.001**
CRP (mg/L), mean ± SD	89.30 ± 58.86	79.38 ± 56.72	109.34 ± 58.25	**< 0.001**
PCT (ng/mL), mean ± SD	1.03 ± 1.01	0.95 ± 1.00	1.20 ± 1.03	**0.003**
Creatinine (mg/dL), mean ± SD	1.36 ± 0.66	1.32 ± 0.65	1.45 ± 0.68	0.094
**Urine microbiology**				
Escherichia coli, n (%)	201 (56.1)	131 (54.4)	70 (59.8)	0.328
*Klebsiella* spp., n (%)	70 (19.6)	51 (21.2)	19 (16.2)	0.271
Other uropathogens*, n (%)	82 (22.9)	55 (22.8)	27 (23.1)	1.000
ESBL-producing organism (urine), n (%)	55 (16.0)	38 (16.5)	17 (15.0)	0.738
Carbapenem-resistant organism (urine), n (%)	5 (1.4)	3 (1.3)	2 (1.8)	0.656
**Antibiotic therapy and outcomes**				
Time to first antibiotic dose, hours, mean ± SD	3.50 ± 1.59	3.97 ± 1.50	2.54 ± 1.33	**< 0.001**
Appropriate empirical therapy within 24 h, n (%)	230 (64.2)	154 (63.9)	76 (65.0)	0.845
Total duration of therapy, days, mean ± SD	10.44 ± 3.24	9.44 ± 2.98	12.50 ± 2.77	**< 0.001**
Source control performed (any), n (%)	62 (17.3)	37 (15.4)	25 (21.4)	0.158
30-day all-cause mortality, n (%)	22 (6.1)	11 (4.6)	11 (9.4)	0.074
Intensive care unit transfer within 72 h, n (%)	40 (11.2)	16 (6.6)	24 (20.5)	**< 0.001**
Clinical failure at day 7, n (%)	23 (6.4)	10 (4.1)	13 (11.1)	**0.012**
Length of stay, days, mean ± SD	7.18 ± 3.50	6.25 ± 3.21	9.09 ± 3.31	**< 0.001**

*Other uropathogens include *Proteus* spp., *Pseudomonas* spp., *Enterococcus* spp., and other less frequent organisms. CA-UTI, community-acquired urinary tract infection; SD, standard deviation; ESBL, extended-spectrum β-lactamase; CRO, carbapenem-resistant organism; CCI, Charlson Comorbidity Index; BBI, Barthel Index; qSOFA, quick Sequential Organ Failure Assessment; SOFA, Sequential Organ Failure Assessment; CRP, C-reactive protein; PCT, procalcitonin; ICU, intensive care unit. Bold values indicate statistical significance (two-sided *p* < 0.05).

### Comorbidity profile and illness severity at presentation

The overall comorbidity burden was modestly higher in the bacteremic group (CCI 2.36 ± 1.40 vs. 2.10 ± 1.35; *p* = 0.098), but several specific conditions were clearly enriched. Diabetes mellitus (41.0% vs. 17.8%; *p* < 0.001), hemodialysis (6.8% vs. 0.0%; *p* < 0.001), dementia (11.1% vs. 3.3%; *p* = 0.003), and solid organ malignancy (9.4% vs. 0.8%; *p* < 0.001) were more frequent among bacteremic patients, whereas other major comorbidities showed no significant between-group differences. Functional status as measured by the BI was comparable (70.9 ± 21.6 vs. 72.8 ± 21.2; *p* = 0.400), but the Pitt bacteremia score at presentation was substantially higher in bacteremic CA-UTI (2.56 ± 1.74 vs. 1.22 ± 1.15; *p* < 0.001), indicating greater acute illness severity.

Clinical findings at admission further supported a more severe presentation in bacteremic CA-UTI (Table 1). Fever > 38°C (70.9% vs. 56.8%; *p* = 0.010), heart rate > 120 beats per minute (26.5% vs. 13.3%; *p* = 0.002), and systolic blood pressure < 90 mmHg (25.6% vs. 7.9%; *p* < 0.001) were all significantly more common in the bacteremia group. Sepsis severity scores also differed markedly: qSOFA ≥ 2 was observed in 12.8% of bacteremic versus 4.6% of non-bacteremic patients (*p* = 0.005), and sepsis defined as SOFA ≥ 2 at admission occurred in 50.4% versus 25.3%, respectively (*p* < 0.001).

### Laboratory findings

Laboratory parameters at presentation were consistent with a more pronounced systemic inflammatory response in bacteremic cases. Compared with non-bacteremic CA-UTI, patients with bacteremia had higher white blood cell counts (9,127 ± 2,218 vs. 7,548 ± 2,085 cells/mm^3^; *p* < 0.001) and neutrophil-to-lymphocyte ratios (7.41 ± 5.23 vs. 5.45 ± 3.69; *p* < 0.001). Inflammatory biomarkers were also significantly elevated, with higher CRP (109.34 ± 58.25 vs. 79.38 ± 56.72 mg/L; *p* < 0.001) and PCT (1.20 ± 1.03 vs. 0.95 ± 1.00 ng/mL; *p* = 0.003) levels in the bacteremic group. Baseline renal function was similar between groups (creatinine 1.45 ± 0.68 vs. 1.32 ± 0.65 mg/dL; *p* = 0.094).

### Microbiology

The urinary microbiological spectrum was dominated by *Escherichia coli* in both bacteremic and non-bacteremic CA-UTI (59.8% vs. 54.4%; *p* = 0.328), followed by *Klebsiella* spp. (16.2% vs. 21.2%; *p* = 0.271) and a mixture of other uropathogens, including *Proteus* spp., *Pseudomonas* spp., *Enterococcus* spp., and less frequent organisms (Table 1). The prevalence of ESBL-producing isolates (15.0% vs. 16.5%; *p* = 0.738) and carbapenem-resistant organisms (1.8% vs. 1.3%; *p* = 0.656) was low and comparable across groups, indicating that the occurrence of bacteremia was not driven by major differences in urinary pathogen distribution or resistance profiles.

### Treatment and clinical outcomes

With respect to treatment, time from presentation to the first antimicrobial dose was significantly shorter in bacteremic CA-UTI (2.54 ± 1.33 vs. 3.97 ± 1.50 h; *p* < 0.001), reflecting earlier recognition and escalation of care. The proportion of patients receiving appropriate empirical therapy within 24 h was high and similar in both groups (65.0% vs. 63.9%; *p* = 0.845). However, bacteremic patients received a longer total duration of antimicrobial therapy (12.50 ± 2.77 vs. 9.44 ± 2.98 days; *p* < 0.001) and had a more prolonged hospital stay (9.09 ± 3.31 vs. 6.25 ± 3.21 days; *p* < 0.001). Overall rates of source control procedures were comparable between bacteremic and non-bacteremic patients (21.4% vs. 15.4%; *p* = 0.158).

Overall 30-day all-cause mortality in the cohort was 6.1% (22/358) (Table 1). Mortality was numerically higher among patients with bacteremic CA-UTI than among those with non-bacteremic infection (9.4% vs. 4.6%), although this difference did not reach statistical significance (*p* = 0.074). Markers of a severe clinical course clustered in the bacteremia group: intensive care unit transfer within 72 h occurred in 20.5% of bacteremic versus 6.6% of non-bacteremic patients (*p* < 0.001), and clinical failure at day 7 was more frequent (11.1% vs. 4.1%; *p* = 0.012). Length of stay was also significantly longer in bacteremic patients, as noted above.

### Predictors of bacteremia

In univariable analyses (Supplementary Table 1), several demographic, comorbidity, functional, and severity variables were associated with bacteremia, including previous hospitalization within 90 days, dementia, solid organ malignancy, lower BI, hemodynamic instability, leukocytosis, elevated neutrophil-to-lymphocyte ratio, and sepsis at admission. In the multivariable logistic regression model assessing risk factors for bacteremia (Table 2), age ≥ 65 years was not independently associated with bloodstream infection (aOR 0.93, 95% CI 0.53–1.63; *p* = 0.801). Independent predictors of bacteremia included female sex (protective; aOR 0.31, 95% CI 0.17–0.55; *p* < 0.001), symptom duration ≤ 3 days (aOR 3.62, 95% CI 2.05–6.40; *p* < 0.001), prior ESBL/CRO colonization or infection within 12 months (aOR 2.88, 95% CI 1.18–7.03; *p* = 0.020), CCI ≥ 2 (aOR 9.27, 95% CI 5.09–16.88; *p* < 0.001), and diabetes mellitus (aOR 3.43, 95% CI 1.85–6.38; *p* < 0.001). Markers of acute physiological derangement and systemic inflammation also remained independently associated with bacteremia, including qSOFA ≥ 2 (aOR 6.61, 95% CI 2.40–18.23; *p* < 0.001), CRP > 100 mg/L (aOR 2.09, 95% CI 1.19–3.67; *p* = 0.011), and PCT ≥ 0.5 ng/mL (aOR 2.01, 95% CI 1.12–3.61; *p* = 0.020).

**TABLE 2:** Risk factors for bacteremia in adults hospitalized with CA-UTI (*n* = 358).

Variable	Unadjusted OR (95% CI)	*p*	Adjusted OR (95% CI)	p (adjusted)
Age ≥ 65 years	1.00 (0.65–1.55)	0.988	0.93 (0.53–1.63)	0.801
Female sex	0.46 (0.29–0.72)	0.001	0.31 (0.17–0.55)	**< 0.001**
Symptom duration ≤ 3 days	2.85 (1.72–4.71)	< 0.001	3.62 (2.05–6.40)	**< 0.001**
Prior ESBL/CRO ≤ 12 months	2.54 (1.25–5.13)	0.010	2.88 (1.18–7.03)	**0.020**
CCI ≥ 2	6.02 (3.71–9.79)	< 0.001	9.27 (5.09–16.88)	**< 0.001**
Diabetes mellitus	2.39 (1.46–3.92)	0.001	3.43 (1.85–6.38)	**< 0.001**
qSOFA ≥ 2	3.16 (1.51–6.65)	0.002	6.61 (2.40–18.23)	**< 0.001**
CRP > 100 mg/L	1.96 (1.25–3.06)	0.003	2.09 (1.19–3.67)	**0.011**
PCT ≥ 0.5 ng/mL	1.54 (0.97–2.45)	0.066	2.01 (1.12–3.61)	**0.020**

All variables shown were included in the multivariable logistic regression model; age and sex were forced to remain in the model regardless of their univariable association with bacteremia. The multivariable model demonstrated good discrimination for bacteremia [c-statistic (area under the receiver-operating characteristic curve, AUROC) 0.83]. OR, odds ratio; aOR, adjusted odds ratio; CI, confidence interval; ESBL, extended-spectrum β-lactamase; CRO, carbapenem-resistant organism; CCI, Charlson Comorbidity Index; qSOFA, quick Sequential Organ Failure Assessment; CRP, C-reactive protein; PCT, procalcitonin. Bold values indicate statistical significance (two-sided *p* < 0.05).

### Bedside bacteremia risk score performance

**FIGURE 1:**
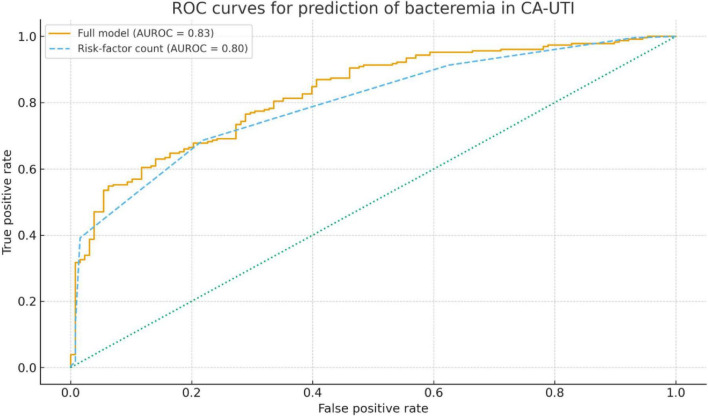
Receiver-operating characteristic (ROC) curves for prediction of bacteremia. Receiver-operating characteristic (ROC) curves for predicting bacteremia in adults hospitalized with community-acquired urinary tract infection. The solid line shows the full multivariable logistic regression model (AUROC 0.83, 95% CI 0.78–0.88), and the dashed line shows the simplified eight-item risk-factor count score (AUROC 0.80, 95% CI 0.74–0.86); the difference between the two AUROCs was not statistically significant (DeLong *p* = 0.32). The diagonal line represents the line of no discrimination.

A simple additive risk-factor count score based on eight binary predictors (male sex, short symptom duration before admission, prior ESBL/CRO colonization or infection, CCI ≥ 2, diabetes mellitus, qSOFA ≥ 2, CRP > 100 mg/L, and PCT ≥ 0.5 ng/mL) showed a marked gradient in the prevalence of bacteremia (Table 3). Among patients with 0–1 risk factor, bacteremia occurred in only 3 of 80 (3.8%). This proportion increased to 47 of 190 (24.7%) among those with 2–3 risk factors and to 66 of 88 (75.0%) in patients with ≥ 4 risk factors. Receiver-operating characteristic curve analysis (Figure 1) demonstrated good discrimination of the full multivariable bacteremia model, with an AUROC of 0.83 (95% CI, 0.78–0.88), and comparable performance of the simplified eight-item risk-factor count score, with an AUROC of 0.80 (95% CI, 0.74–0.86); the difference between the two AUROCs was not statistically significant (DeLong p = 0.32).

**TABLE 3:** Bacteremia rate according to the number of risk factors.

Risk group (number of risk factors)	*n*	Bacteremia, n (%)
0–1	80	3 (3.8)
2–3	190	47 (24.7)
≥ 4	88	66 (75.0)

Risk factors included in the eight-item score were: male sex, symptom duration ≤ 3 days before admission, prior ESBL/CRO colonization or infection, CCI ≥ 2, diabetes mellitus, qSOFA ≥ 2, CRP > 100 mg/L, and PCT ≥ 0.5 ng/mL.

### Mortality analyses

**FIGURE 2:**
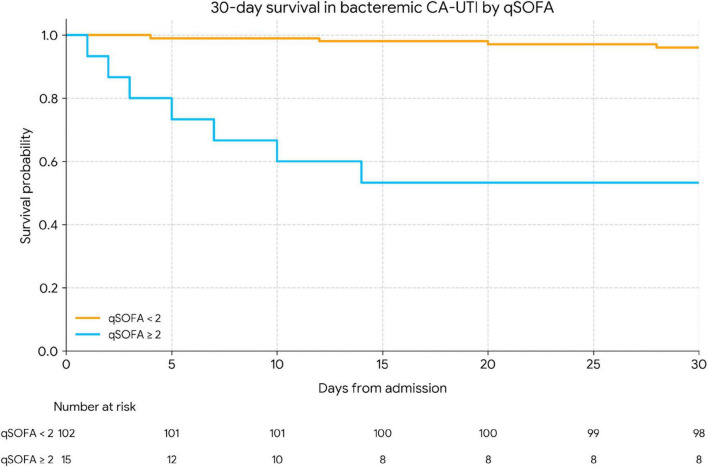
Kaplan–Meier 30-day survival in bacteremic CA-UTI stratified by Qsofa. Kaplan–Meier curves for 30-day survival among adults with bacteremic community-acquired urinary tract infection, stratified by qSOFA at presentation (qSOFA < 2 vs. qSOFA ≥ 2). Survival was significantly worse in patients with qSOFA ≥ 2 (log-rank *p* < 0.001). The number of patients at risk in each group at selected time points is shown below the x-axis.

In the bacteremic CA-UTI subgroup (*n* = 117), 30-day all-cause mortality was 9.4% (11/117) (Table 1). Univariable analysis identified qSOFA ≥ 2 as a strong predictor of death (unadjusted OR 15.94, 95% CI 5.11–49.74; *p* < 0.001), whereas age ≥ 65 years and CCI ≥ 2 showed non-significant associations with wide confidence intervals (Table 4). In an exploratory multivariable model restricted to bacteremic patients and including age ≥ 65 years, CCI ≥ 2, and qSOFA ≥ 2, qSOFA ≥ 2 remained the only independent predictor of 30-day mortality (aOR 18.84, 95% CI 5.50–64.48; *p* < 0.001), while age ≥ 65 years (aOR 2.26, 95% CI 0.71–7.15; *p* = 0.166) and CCI ≥ 2 (aOR 1.30, 95% CI 0.35–4.75; *p* = 0.695) were not independently associated with death. However, because only 11 deaths occurred in this subgroup, the events-per-variable ratio was low, confidence intervals were wide, and these estimates should be interpreted cautiously as hypothesis-generating. Accordingly, the adjusted odds ratios in Table 4 should be viewed as imprecise and primarily descriptive of directionality rather than definitive effect estimates. Kaplan–Meier survival curves confirmed significantly worse 30-day survival among bacteremic patients with qSOFA ≥ 2 compared with those with qSOFA < 2 (Figure 2).

**TABLE 4:** Predictors of 30-day mortality among adults with bacteremic community-acquired urinary tract infection (*n* = 117).

Variable	Unadjusted OR (95% CI)	*p*	Adjusted OR (95% CI)	p (adjusted)
Age ≥ 65 years	1.41 (0.56–3.56)	0.469	2.26 (0.71–7.15)	0.166
CCI ≥ 2	1.84 (0.57–5.90)	0.306	1.30 (0.35–4.75)	0.695
qSOFA ≥ 2	15.94 (5.11–49.74)	< 0.001	18.84 (5.50–64.48)	**< 0.001**

Because only 11 deaths occurred in the bacteremic subgroup, this multivariable model has a low events-per-variable ratio and is at risk of overfitting; the results should therefore be interpreted as exploratory. CA-UTI, community-acquired urinary tract infection; CCI, Charlson Comorbidity Index; qSOFA, quick Sequential Organ Failure Assessment; OR, odds ratio; CI, confidence interval. Bold values indicate statistical significance (two-sided *p* < 0.05).

In the overall CA-UTI cohort, predictors of 30-day mortality are summarized in Table 5. In univariable analyses, bacteremia, CCI ≥ 2, and qSOFA ≥ 2 were all associated with increased risk of death. After adjustment, both bacteremia (aOR 9.74, 95% CI 2.64–35.96; *p* = 0.001) and qSOFA ≥ 2 (aOR 21.82, 95% CI 7.32–65.05; *p* < 0.001) remained independent determinants of 30-day mortality, whereas CCI ≥ 2 lost statistical significance (aOR 1.29, 95% CI 0.41–4.02; *p* = 0.665). Age ≥ 65 years showed a non-significant trend toward higher mortality (aOR 2.78, 95% CI 0.98–7.90; *p* = 0.056). Overall, these findings indicate that bacteremia and, more prominently, acute physiological derangement captured by qSOFA ≥ 2 identify patients with CA-UTI at high risk for short-term adverse outcomes, although the wide confidence intervals and limited number of deaths necessitate cautious interpretation of the effect sizes. Given the modest number of deaths overall, the multivariable mortality estimates in Table 5 should be interpreted conservatively, with emphasis on risk stratification rather than precise quantification of effect sizes.

**TABLE 5:** Predictors of 30-day mortality in the overall CA-UTI cohort (*n* = 358).

Variable	Unadjusted OR (95% CI)	*p*	Adjusted OR (95% CI)	p (adjusted)
Bacteremia	12.76 (4.29–37.98)	< 0.001	9.74 (2.64–35.96)	**0.001**
Age ≥ 65 years	1.56 (0.69–3.49)	0.282	2.78 (0.98–7.90)	0.056
CCI ≥ 2	3.59 (1.47–8.77)	0.005	1.29 (0.41–4.02)	0.665
qSOFA ≥ 2	20.35 (8.23–50.34)	< 0.001	21.82 (7.32–65.05)	**< 0.001**

Variables with *p* < 0.10 in univariable analysis and/or judged to have strong clinical relevance were considered for inclusion in the multivariable logistic regression model. The final multivariable model included bacteremia, age ≥ 65 years, CCI ≥ 2, and qSOFA ≥ 2. OR, odds ratio; aOR, adjusted odds ratio; CI, confidence interval; CCI, Charlson Comorbidity Index; qSOFA, quick Sequential Organ Failure Assessment. Bold values indicate statistical significance (two-sided *p* < 0.05).

## Discussion

In this study, we identified a distinct high-risk phenotype for bacteremic CA-UTI characterized by male sex, specific comorbidities, fulminant symptom onset, and a profound systemic inflammatory response. Although bacteremia was present in one-third of our cohort, consistent with the 24–33% prevalence reported in comparable tertiary care settings (1, 18), our findings provide a more nuanced perspective on the role of age. Unlike population-based surveillance studies that depict a linear correlation between advancing age and bacteremia risk (2, 6), age did not emerge as an independent predictor in our multivariate model. This divergence highlights a critical distinction in hospitalized cohorts: age likely functions as a proxy for accumulated frailty and immune senescence rather than as an independent driver of bloodstream invasion. In our study, the specific nature of the underlying pathology (e.g., diabetes, malignancy) outweighed chronological age, suggesting that biological reserve is a more accurate determinant of invasiveness than demographic age alone.

A key finding of our analysis is that bacteremic patients presented with a significantly shorter duration of symptoms than non-bacteremic cases. Rather than reflecting mild disease, this inverse relationship likely indicates failure of early mucosal containment and a more aggressive host–pathogen interaction. In contrast to the more gradual course of localized cystitis, bacteremic cases appear to follow a more rapidly progressive trajectory, with escalation from lower urinary tract symptoms to high fever and hemodynamic compromise, prompting earlier presentation to the emergency department (1, 24). This pattern is biologically plausible: a blunted or delayed innate immune response at the uroepithelial surface may permit rapid bacterial proliferation and translocation into the bloodstream, particularly in the presence of virulence factors that enhance adhesion, immune evasion, and serum resistance. The association of this phenotype with male sex further supports a mechanistic link. Although females generally have a higher incidence of uncomplicated UTI, males in our cohort demonstrated a substantially higher propensity for bacteremic progression. This is consistent with the concept that urinary tract infections in males are intrinsically complicated due to anatomical factors, including prostatic hypertrophy and urinary retention, which promote bacterial persistence and impair adequate urinary washout (10). Moreover, translational data suggest that males may mount less robust and slower local mucosal immune responses than females, thereby facilitating a quicker hematogenous spread despite shorter symptom duration (10, 25). Taken together, our data suggest that brief symptom duration in the presence of systemic features should be interpreted as a potential marker of a more invasive course rather than as a reassuring feature of mild disease.

Our model confirms that bacteremia risk is driven by the severity of host compromise. The persistence of DM as a robust independent risk factor (aOR 3.43, 95% CI 1.85–6.38) warrants specific attention. This association likely extends beyond simple hyperglycemia; it reflects a complex interplay of diabetic cystopathy (urinary stasis) and distinct immune defects, particularly impaired neutrophil chemotaxis and phagocytosis, which facilitate the breach of the uroepithelial barrier (26, 27). Similarly, the strong association with solid-organ malignancy and recent healthcare exposure points to a “vulnerable host” phenotype. In these patients, the urinary tract frequently serves as a portal of entry for opportunistic pathogens, underscoring the need for heightened vigilance in oncologic patients presenting with even subtle urinary symptoms (28, 29). Notably, the comorbidity profile of our cohort, characterized by diabetes, chronic kidney disease, dementia, and solid-organ malignancy, is highly consistent with that reported in contemporary CA-UTI and bacteremic sepsis cohorts from other tertiary-care hospitals. (30, 31). This heterogeneity supports the external validity of our findings and suggests that, despite the single-center design, our results are likely to be generalizable to similar inpatient populations in settings with a comparable case mix and antimicrobial resistance ecology.

In addition, our study highlights the intersection between antimicrobial resistance and clinical severity. A history of ESBL/CRO colonization or infection within the previous year was a strong predictor of bacteremia (aOR 2.88, 95% CI 1.18–7.03). Notably, the distribution of urinary pathogens and their antimicrobial resistance profiles were broadly comparable between bacteremic and non-bacteremic CA-UTI cases in our cohort. This finding suggests that a history of prior ESBL/CRO infection may represent a broader phenotype encompassing host vulnerability and prior healthcare exposure, rather than merely indicating increased antimicrobial resistance at the index episode. This observation suggests that resistance is not only a therapeutic challenge but may also signal an increased risk of invasive disease. Patients with prior healthcare exposure and resistant colonization are more likely to receive inappropriate initial empirical therapy and may harbor pathogens with enhanced virulence potential (32, 33). The coexistence of delayed effective treatment and host vulnerability may facilitate progression from localized infection to bacteremia. These findings support the incorporation of prior colonization history into risk stratification algorithms at triage. In our cohort, ESBL-producing uropathogens accounted for 16% of isolates, which places our setting within the intermediate range reported for community-acquired UTIs worldwide. Contemporary data indicate that ESBL-producing E. coli in CA-UTIs ranges from approximately 3–48% across regions, with reported rates of 6.3% in uncomplicated and 17.4% in complicated infections in Türkiye (34), 3.3% in community-onset infections and up to 12.1% among nursing-home residents in France (35, 36), and 37.2% in Chinese cohorts (37); in the United States, 36.8% of ESBL-positive E. coli infections now originate in the community, and more than half occur in patients without traditional healthcare-associated risk factors (38). This suggests that our setting does not represent an extreme-resistance hotspot and that the results are likely to apply to populations with similar or moderately lower ESBL burdens, while still aligning with the worldwide trend of increasing ESBL prevalence.

We demonstrate the utility of integrating physiological scores with inflammatory biomarkers for early risk stratification. The strong independent association of qSOFA ≥ 2 with bacteremia suggests that this simple bedside tool effectively captures the threshold of sepsis-defining organ dysfunction (39). In addition, both elevated CRP (> 100 mg/L) and PCT (≥ 0.5 ng/mL) were independently associated with bacteremia, supporting their role as markers of a higher systemic inflammatory and infectious burden. PCT is generally regarded as more specific than CRP for systemic bacterial infections and sepsis, particularly in distinguishing bacterial from viral or non-infectious inflammatory states, and our findings are broadly consistent with this literature (40–43). However, we did not formally compare the discriminatory performance of CRP and PCT, and our data should therefore not be interpreted as demonstrating diagnostic superiority of one biomarker over the other. Clinically, a markedly elevated PCT level in a patient with UTI symptoms may justifiably raise the index of suspicion for bacteremia and prompt closer monitoring and timely escalation of therapy. In contrast, low PCT values could support more conservative antibiotic strategies in carefully selected patients.

The clinical impact of bacteremia in our cohort extended beyond the acute phase, resulting in increased utilization of healthcare resources. Bacteremic patients had an approximately threefold higher rate of ICU admission and a longer length of stay. The higher rate of 7-day clinical failure in this group reflects the difficulty of eradicating invasive infections, particularly when complicated by the resistance patterns discussed above. These findings suggest that the bacteremic subgroup disproportionately drives the economic and operational cost of CA-UTI. Therefore, early identification using the risk factors we identified (male sex, DM, qSOFA, PCT) could facilitate earlier aggressive interventions (e.g., source control, targeted antibiotics), potentially mitigating these downstream costs and improving clinical success rates (19, 44).

From a prognostic perspective, two complementary observations emerged. In the overall CA-UTI cohort, both bacteremia and qSOFA ≥ 2 were independently associated with 30-day mortality, with qSOFA exerting a more substantial relative effect, highlighting the central role of acute organ dysfunction. This pattern is consistent with findings from severe sepsis cohorts, in which short-term survival correlates more closely with the extent of organ failure than with microbiological parameters alone (45–47). In contrast, within the subset of patients with bacteremic CA-UTI, qSOFA ≥ 2 remained the only independent predictor of death. In contrast, age and comorbidity burden did not retain statistical significance in the exploratory multivariable model. Taken together, these data suggest that, while bacteremia identifies a higher-risk group at the time of presentation, the short-term prognosis is primarily determined by the severity of organ dysfunction that has occurred with bacteremia. Clinically, this supports a risk-stratification strategy that considers both bacteremia and qSOFA, but prioritizes early recognition and aggressive management of organ dysfunction in patients with elevated qSOFA scores.

Our study has limitations inherent to its single-center, retrospective design, which precludes causal inference and remains vulnerable to selection bias and residual confounding despite multivariable adjustment. Because we focused on hospitalized adults and excluded mild outpatient CA-UTI, the observed prevalence of bacteremia, sepsis, and short-term adverse outcomes may be higher than in unselected community cohorts. In addition, center-specific antimicrobial resistance ecology and care pathways in Türkiye may limit generalizability to settings with different patient populations, diagnostic practices, and ecological profiles. Although the sample size was adequate for the primary endpoints, the mortality analyses were likely underpowered to detect smaller effect sizes within subgroups, such as the borderline association observed with impaired functional status. Moreover, the overall number of deaths was modest, with 22 events in the full cohort and 11 deaths in the bacteremic subgroup, resulting in a low events-per-variable ratio. This increases the risk of overfitting and may yield potentially unstable adjusted estimates in Tables 4, 5. These findings should therefore be interpreted cautiously as exploratory and hypothesis-generating. Several predictors were operationalized using clinically motivated binary thresholds to facilitate bedside interpretability and risk-score construction. However, dichotomization can reduce statistical power and obscure dose–response or non-linear relationships, and sensitivity analyses using continuous or spline-based specifications were not performed. Finally, the eight-item bacteremia risk score was derived and evaluated within the same cohort without resampling-based internal validation, such as bootstrapping or cross-validation. Consequently, its apparent discrimination may be optimistic, and the reported AUROC should be interpreted as derivation-sample performance that may overestimate true performance in independent populations. Future multicenter studies should externally validate discrimination and calibration, assess clinical utility, and evaluate transportability across diverse healthcare systems and epidemiological contexts.

## Conclusion

In conclusion, our data convey three complementary take-home messages. First, chronological age alone is a poor discriminator of risk in hospitalized adults with CA-UTI; instead, specific vulnerability markers—including diabetes, higher comorbidity burden, prior ESBL/CRO colonization or infection, and acute physiological derangement—more accurately define the phenotype prone to bacteremic progression. Second, in the overall cohort, both bacteremia and a qSOFA score of 2 or higher were independently associated with 30-day mortality. Among patients with bacteremic CA-UTI, mortality risk was primarily stratified by the severity of organ dysfunction (qSOFA ≥ 2). Third, we derived a simple additive eight-item risk score based on readily available clinical variables that predicted bacteremia with good internal discrimination (AUROC 0.83 for the whole model and 0.80 for the simplified score); however, this score should be regarded as exploratory and requires external validation in independent cohorts before it can be adopted for routine triage decisions. Taken together, these findings support a pragmatic approach in which clinicians prioritize comorbidity profile, history of resistant colonization, and early markers of organ dysfunction over age alone or blood culture status when triaging and managing adults hospitalized with CA-UTI.

## Data Availability

The raw data supporting the conclusions of this article will be made available by the authors, without undue reservation.
