# Supplementary material for: Beyond bacteremia: clinical phenotypes and determinants of mortality in hospitalized adults with community-acquired urinary tract infection
Source: Front Med (Lausanne). 2026 Feb 11;13:1754663. doi: 10.3389/fmed.2026.1754663 (PMC12932189; doi:10.3389/fmed.2026.1754663)
Supplement: Supplementary file 1 [file Table_1.docx]

**SUPPLEMENTARY TABLE S1 Univariable logistic regression analysis of risk factors for bacteremia in adults hospitalized with community-acquired urinary tract infection (n = 358)**

| **Variable** | **OR (95% CI)** | **p value** |
| --- | --- | --- |
| Long-term care facility residence | 1.26 (0.69–2.29) | 0.529 |
| Recent healthcare exposure (≤90 days) | 1.30 (0.76–2.21) | 0.336 |
| Previous hospitalization ≤90 days | 1.76 (1.03–2.99) | 0.037 |
| Obstructive uropathy | 0.91 (0.38–2.16) | 0.829 |
| Urinary tract anatomical anomalies | 0.88 (0.22–3.47) | 0.855 |
| Prostate disease (BPH/prostatitis) | 1.56 (0.69–3.52) | 0.280 |
| Indwelling urinary catheter | 1.12 (0.65–1.92) | 0.680 |
| Double-J stent | 1.20 (0.63–2.27) | 0.585 |
| Recent urological instrumentation ≤30 d | 0.70 (0.30–1.61) | 0.395 |
| Urologic surgery ≤12 months | 1.03 (0.52–2.05) | 0.924 |
| Chronic kidney disease* | 0.89 (0.48–1.62) | 0.697 |
| Ischemic heart disease | 1.33 (0.76–2.31) | 0.320 |
| Heart valve disease | 1.29 (0.52–3.20) | 0.586 |
| Chronic liver disease | 0.95 (0.35–2.56) | 0.916 |
| Dementia | 3.64 (1.46–9.05) | 0.005 |
| Solid organ malignancy | 12.40 (2.70–56.92) | 0.001 |
| Urologic tumor | 2.11 (0.60–7.43) | 0.246 |
| Solid organ / renal transplant | 2.07 (0.13–33.37) | 0.608 |
| Immunosuppression (any) | 1.85 (0.66–5.24) | 0.245 |
| Chronic systemic steroid use | 1.30 (0.42–4.06) | 0.652 |
| Barthel Index <40 | 4.48 (2.01–9.98) | <0.001 |
| Barthel Index 40–60 | 1.30 (0.76–2.21) | 0.335 |
| Barthel Index >60 | 0.82 (0.51–1.32) | 0.408 |
| Fever >38°C | 1.85 (1.15–2.98) | 0.011 |
| Heart rate >120 bpm | 2.35 (1.35–4.10) | 0.002 |
| Systolic blood pressure <90 mmHg | 4.03 (2.15–7.53) | <0.001 |
| Sepsis at admission (SOFA ≥2) | 3.00 (1.89–4.78) | <0.001 |
| Leukocytosis >12,000/mm³ | 5.39 (1.85–15.70) | 0.002 |
| Neutrophil-to-lymphocyte ratio ≥7 | 1.67 (1.04–2.67) | 0.033 |
| Hemoglobin (g/dL) | 1.01 (0.88–1.16) | 0.905 |
| Thrombocytopenia <100,000/mm³ | 0.77 (0.20–2.94) | 0.698 |
| Creatinine (mg/dL) | 1.33 (0.95–1.87) | 0.098 |

***Abbreviations:*** *OR, odds ratio; CI, confidence interval; SOFA, Sequential Organ Failure Assessment; BPH, benign prostatic hyperplasia.*

** Chronic kidney disease defined as estimated glomerular filtration rate (eGFR) <60 mL/min/1.73 m².*

***Note:*** *Variables already reported in Table 2 (age ≥65 years, female sex, symptom duration before admission, prior ESBL/CRO colonization or infection, CCI ≥2, diabetes mellitus, qSOFA ≥2, C-reactive protein >100 mg/L, and procalcitonin ≥0.5 ng/mL) and variables with unstable estimates due to sparse data (hemodialysis) were omitted from this supplementary table to avoid redundancy and model separation issues.*
